# MicroRNA-1246 regulates macrophage lipid metabolism and is involved in resistance to human *Mycobacterium tuberculosis* infection

**DOI:** 10.1371/journal.ppat.1014454

**Published:** 2026-10-13

**Authors:** Ashutosh Tripathi, Venkata Sanjeev Kumar Neela, Rajesh Kumar Radhakrishnan, Kamakshi Prudhula Devalraju, Anvesh Kumar Bogam, Tanmoy Mukherjee, Varalakshmi Mallidi, Abhinav Vankayalapati, Mohammed Soheb Sadath Ansari, Carlos Arana, Sindhu Joshi, Karan P. Singh, Vijaya Lakshmi Valluri, Ramakrishna Vankayalapati, Deepak Tripathi

**Affiliations:** 1 Center for Global Infectious Disease Research, Seattle Children’s Research Institute, Seattle, Washington, United States of America; 2 Immunology and Molecular Biology Department, Bhagwan Mahavir Medical Research Centre, Hyderabad, India; 3 Division of Infectious Diseases, Allergy and Immunology, Department of Internal Medicine, Saint Louis University School Medicine, St. Louis, Missouri, United States of America; 4 Center for Biomedical Research, The University of Texas Health Science Center at Tyler, Tyler, Texas, United States of America; 5 Department of Immunology, UT Southwestern Medical Center, Dallas, Texas, United States of America; 6 Department of Epidemiology and Biostatistics, The University of Texas at Tyler School of Medicine, Health Science Center, Tyler, Texas, United States of America; 7 Department of Medicine, Tulane University School of Medicine, New Orleans, Louisiana, United States of America; Portland VA Medical Center, Oregon Health and Science University, UNITED STATES OF AMERICA

## Abstract

We determined whether microRNAs (miRNAs) regulate resistance to *Mycobacterium tuberculosis* (Mtb) infection in healthy household contacts (HHCs) of patients with tuberculosis (TB). As shown in our previously published study, we recruited 460 HHCs from 443 index cases of active pulmonary TB, with 293 (64.8%) remaining IGRA-negative throughout a two-year follow-up. Whole-transcriptome sequencing at baseline (0 months) and follow-up (24 months) and confirmation studies identified miRNA-1246 (downregulated) and miRNA-125b (upregulated) as potential candidates for the development of resistance in nonconverters compared with converters and progressors. Mechanistic studies using proteomics and computational analysis revealed that miRNA-1246 inhibitor reduce Mtb growth in macrophages by regulating lipase activity, specifically through targeting alpha/beta hydrolase domain-containing protein 2 (ABHD2). Inhibition of miRNA-1246 increased glycolytic activity and promoted a metabolic shift in macrophages conducive to pathogen clearance, suggesting its critical role in metabolic regulation and resistance to tuberculosis. These findings highlight potential miRNA-based therapeutic strategies to enhance immunity against Mtb.

## Author summary

Tuberculosis (TB), caused by *Mycobacterium tuberculosis* (Mtb), remains a major global health problem. Interestingly, many household contacts of TB patients remain uninfected despite prolonged exposure, suggesting the presence of natural protective immune mechanisms. In this study, we investigated whether microRNAs (miRNAs), small regulatory RNA molecules that control gene expression, contribute to resistance against Mtb infection. By longitudinally following household contacts of TB patients for two years, we identified distinct miRNA expression patterns in individuals who remained persistently uninfected compared with those who developed latent or active TB. Among the identified candidates, miRNA-1246 was consistently reduced in resistant individuals. Functional studies in human macrophages demonstrated that inhibition of miRNA-1246 restricted Mtb growth by enhancing lipase activity, reducing intracellular lipid accumulation, and increasing glycolytic metabolism. Mechanistically, miRNA-1246 regulated the lipid-metabolism-associated protein ABHD2, which contributed to limiting bacterial survival within macrophages. These findings reveal a previously unrecognized role for miRNA-1246 in coordinating macrophage metabolism and antimicrobial responses during Mtb infection. Our study identifies miRNA-1246 as a potential biomarker and therapeutic target for enhancing host resistance against tuberculosis.

## Introduction

*Mycobacterium tuberculosis* (Mtb) caused an estimated 1.23 million deaths and 10.7 million incident cases of tuberculosis (TB) worldwide in 2024, making TB the world’s leading cause of death from a single infectious agent and one of the leading causes of death globally. [1]. Household contacts (HHCs) of TB patients are a high-risk group for developing latent or active TB. A significant number (>50%) of household contacts of TB patients remain uninfected and do not develop latent or active TB, although this varies with exposure intensity and host susceptibility [2–4]. Recent studies have demonstrated that metabolites, various immune cell populations, signaling molecules and gene polymorphisms play important roles in resistance against Mtb infection in individuals who never develop latent or active TB [5]. There is limited information available about the role of microRNAs (miRNAs) in the resistance or development of latent or active TB.

miRNAs are conserved noncoding RNAs that regulate more than 60% of eukaryotic genes post transcriptionally by binding to complementary 3’-UTR regions of target mRNAs [6], influencing gene expression through destabilization and translational inhibition [7]. miRNA profiles are closely associated with various pathophysiological conditions [8]. miRNAs regulate diverse cellular functions and are implicated in various diseases, including cancer, cardiovascular conditions, diabetes and tuberculosis (TB) [7,9–12]. In Mtb infection, miRNA-125a inhibits autophagy activation and antimicrobial responses [13] while, miRNA125b helps in TNF-α block [14]. miRNAs such as miRNA-155-5p and miRNA-29-3p impair immune responses by targeting IFN-γ and TNF-α, whereas miRNA-21-5p and miRNA-146a-5p attenuate inflammatory responses, thereby influencing disease progression. [15]. The miRNA-29 inhibits immune responses to BCG [16] and Mtb infection and miRNA-223 is expressed in the blood and lung parenchyma of TB patients and controls susceptibility to tuberculosis, regulating lung neutrophil recruitment [17]. Compared with LTBI+ household contacts, TB patients have decreased miRNA-21, 26a, 29a, and 142-3p levels, and miRNA profiles in serum can be used to distinguish active and latent Mtb infection [18]. miRNA-155-5p inhibits the survival of intracellular mycobacteria through different mechanisms, including promoting phagosome maturation and autophagy induction [19], suggesting that miRNAs play important roles in the regulation of immune responses to Mtb infection.

It is not known whether changes in miRNAs impact the development of resistance to TB in a large group of people who have either known Mtb exposure but never developed LTBI or active TB. In the present study, we followed 460 healthy (no comorbid conditions) HHCs of pulmonary TB patients for 2 years at 4-month intervals and determined the miRNA profile of the nonconverters, LTBI converters and those who developed active TB. We also determined that the expression of certain miRNAs in nonconverter HHCs (those who never developed active or latent TB) enhances innate immune responses mediated by macrophages to provide protection against Mtb. We found that miRNA-1246 in nonconverters regulates the lipid metabolism of Mtb-infected macrophages and resistance to Mtb infection.

## Results

### Nonconverters exhibit distinct miRNA expression profiles

We longitudinally followed a cohort of 460 latent tuberculosis infection negative healthy household contacts (LTBI- HHCs) for 2 years, with assessments conducted at 4 month intervals (study design presented in Fig 1A) [20]. As previously reported in Fig 1A of the published work [20], 96 individuals (21.1%) converted to interferon-γ release assay (IGRA)-positive status during the follow-up period. In contrast, despite similar levels of exposure, 293 HHCs (64.8%) consistently remained IGRA negative until the end of the 2-year follow-up and were classified as nonconverters throughout the study duration. Additionally, eight LTBI negative HHCs who progressed to active tuberculosis (ATB) disease were included in this study to enable a comparative analysis between LTBI- HHCs who remain LTBI negative (nonconverters) and those who either converted to IGRA-positive status (converters) or progressed to active TB disease (progressors).

**Fig 1:**
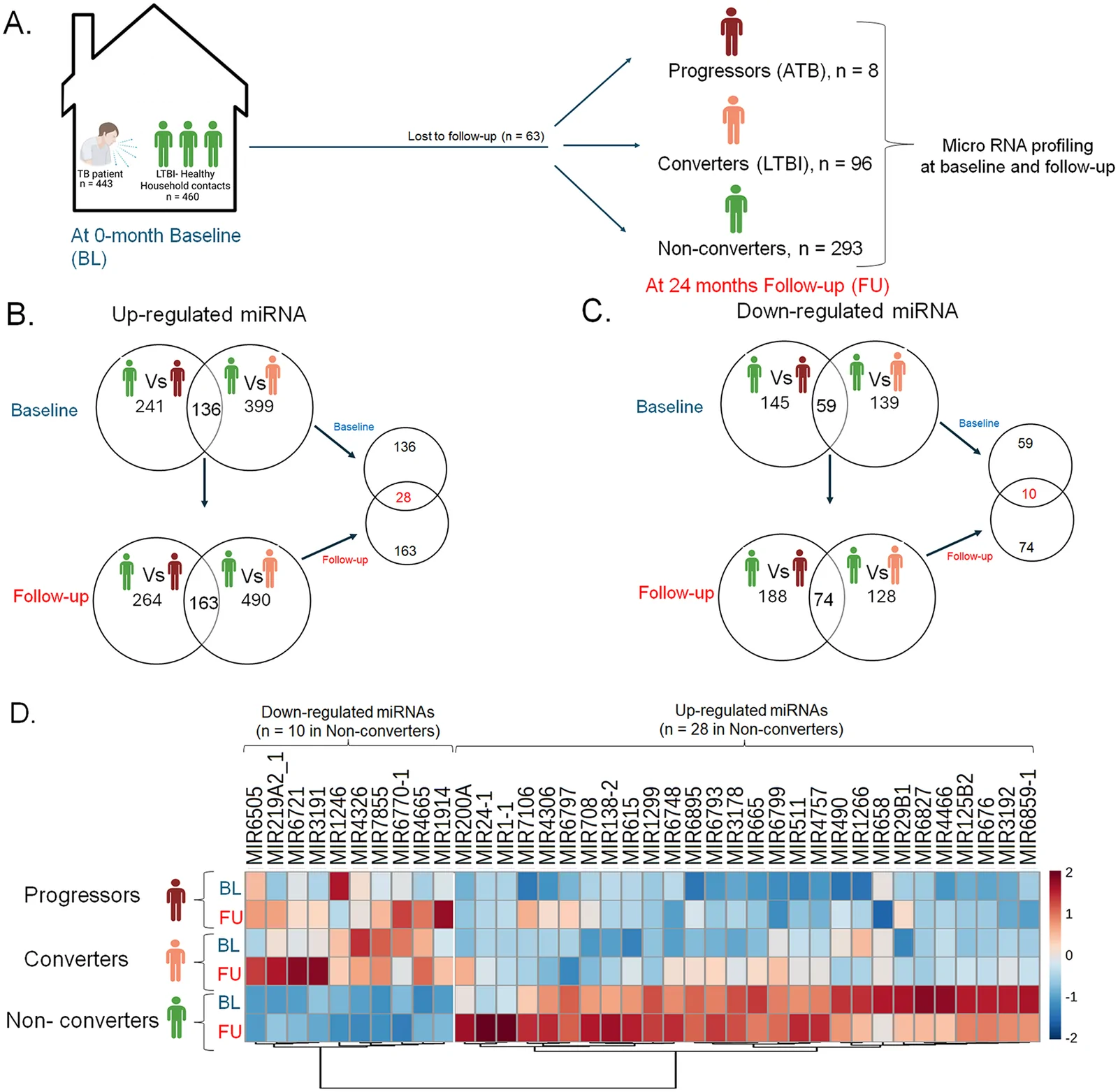
Study design showing donor recruitment, latent or active TB development and miRNA profile at baseline and follow-up. **(A)** Flowchart illustrates the recruitment process of 460 healthy household contacts (HHCs) identified from 443 index cases of active tuberculosis (ATB). This recruitment schematic and details were already shown in one of published study [20]. All HHCs were tested negative for latent tuberculosis infection (LTBI-) using the interferon-gamma release assay (IGRA) at base line and were followed every four months for a period of two years. PBMCs were isolated from age-matched, epidemiologically risk-matched, healthy nonconverters and converters (without comorbid conditions or immunosuppressive drug use) at baseline (during study enrollment, when all participants were LTBI-negative) and after 24 months of follow-up. **(B and C)** miRNA expression profiles were analyzed by RNA sequencing from PBMCs, identifying commonly up-regulated miRNAs **(B)** and down-regulated miRNAs **(C)** in nonconverters compared to converters and progressors at both baseline and follow-up. **(D)** Heatmap showing the expression patterns of the up-regulated and down-regulated miRNA candidates in nonconverters, converters, and progressors (n = 3/group). The *P*-values were determined by 1-way ANOVA with Tukey’s multiple comparisons test. Created in BioRender. Tripathi, D. (2026) https://BioRender.com/e7du35z.

We previously reported RNA sequencing data for two groups of donors - nonconverters and LTBI converters (GSE211566). In the current study, we have expanded this dataset to include individuals who progressed to ATB. We analyzed whole-transcriptome data to identify commonly upregulated and downregulated miRNA candidates in nonconverters relative to LTBI converters and ATB progressors. At baseline, we identified 241 upregulated and 145 downregulated miRNAs in nonconverters compared with ATB progressors and 399 upregulated and 139 downregulated miRNAs compared to LTBI converters. At follow-up, there were 264 upregulated and 188 downregulated miRNAs in nonconverters versus progressors, as well as 490 upregulated and 128 downregulated miRNAs in nonconverters compared with converters (Fig 1B and 1C).

We identified 28 commonly upregulated (Fig 1B and 1D) and 10 downregulated (Fig 1C and 1D) miRNAs in nonconverters compared with both converters and progressors at baseline and follow-up. Additionally, principal component analysis (PCA) of these 38 miRNAs clearly distinguished between the nonconverter group and the converter and ATB progression groups (S1 Fig).

### Differential expression of miR-1246 and miR-125b in Mtb antigen-stimulated PBMCs of nonconverters at baseline and follow-up

We identified 38 differentially regulated miRNAs through computational analysis of transcriptomic data from nonconverters compared with both converters and progressors common in both the timelines at baseline and follow-up (Fig 1B–1D). We validated miRNA expression by real-time PCR analysis using stimulated PBMCs in the presence of Mtb antigens (ESAT-6 and CFP-10) from an additional 11 nonconverters, 6 LTBI converters, and 6 individuals with ATB progressors at baseline and during follow-up. Among the 38 miRNAs, only 8 could be detected across all the samples in the Mtb antigen-stimulated PBMCs (S2A and S2B Fig). Among these, two miRNAs, hsa-miRNA-1246 (hereafter referred to as miRNA-1246) and hsa-miRNA-125b (hereafter referred to as miRNA-125b), were successfully validated and aligned with the RNA-seq results shown in Fig 2A and 2B. Despite the robust expression of the remaining 6 miRNAs, no significant fold change was observed in nonconverters compared with converters and progressors. miRNA-1246 was significantly downregulated, whereas miRNA-125b was upregulated in nonconverter antigen-stimulated PBMCs compared with LTBI and ATB antigen-stimulated PBMCs at both baseline and follow-up (Fig 2A and 2B). In LTBI+ HHCs, miRNA-1246 expression in unstimulated PBMCs was increased at follow-up compared to baseline (S2B Fig). To determine the difference between nonconverters and IGRA-negative healthy individuals (those who were never exposed to TB), we analyzed the expression of these miRNANAs in healthy controls. Nonconverter HHCs had increased expression of miRNA-125b and decreased expression of miRNA-1246 (Fig 2A and 2B). Silencing miRNA-125b-5p protects human macrophages from Mtb infection by enhancing apoptosis and suppressing inflammation through DRAM2 regulation [21]. Various cancer cells express miRNA-1246 [22–26], which is also associated with inflammatory bowel disease. SARS-CoV-2 infection increases miRNA-1246 expression [27]. Macrophages play crucial roles in innate immunity against Mtb, and miRNA-1246, which is highly expressed in macrophages, can modulate their function [26,28,29]. No information is available about the role of miRNA-1246 during Mtb infection. We further determined the role of miRNA-1246 in macrophage-mediated immune responses against Mtb infection.

**Fig 2:**
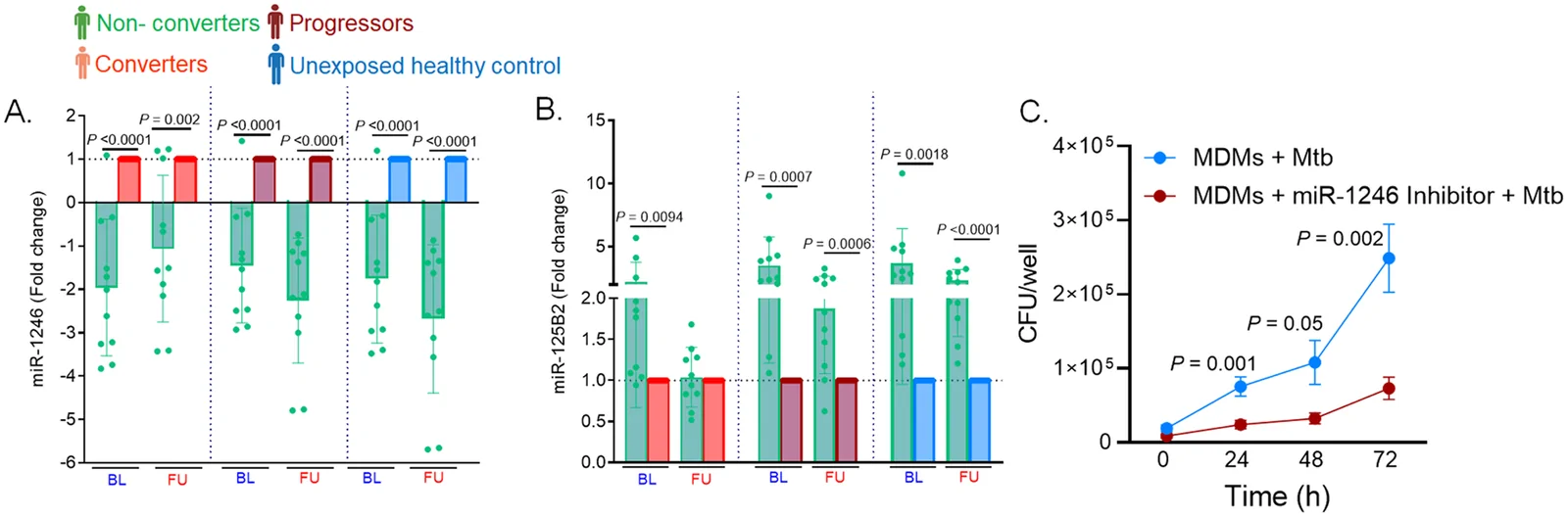
Nonconverters exhibit differential expressions of miR-1246 and miR-125b miRNAs. Real-time PCR analysis of Mtb antigen-stimulated PBMCs from non-converters, LTBI converters and individuals with ATB at baseline and follow-up confirmed differential expression. **(A and B)** TaqMan-based qRT-PCR validation in non-converters (n = 11) compared to converters (n = 6) and progressors (n = 6) in an extended cohort of peripheral blood mononuclear cells (PBMCs). miRNA-191 was used as the housekeeping reference gene to normalize the data. Data shown as mean ± SD and the *P*-value shown in each graph were determined by using a 2-tailed unpaired t-test. **(C)** Intracellular survival of Mtb was assessed in PBMC-derived CD14 + macrophages (MDM) at an MOI of 1:5, pre-transfected with miRNA-1246 inhibitor, and along with a negative control (scrambled RNA). Data is presented as the standard deviation of the mean for the number of CFUs per well. The *P*-value shown in each graph was determined by using a 2-tailed unpaired t-test. Created in BioRender. Tripathi, D. (2026) https://BioRender.com/4obs9ur.

### miR-1246 inhibition reduces Mtb growth in MDMs

To determine the role of miRNA-1246 in Mtb growth, monocyte-derived macrophages (MDMs) were transfected with an inhibitor of miRNA-1246 or a negative control for 48 hours. Quantitative reverse transcription PCR (qRT-PCR) analysis revealed a reduction in miRNA-1246 expression levels of up to 91.1 percent in the inhibitor-treated cells, whereas miRNA-1246 was overexpressed by more than 3000 percent in the negative control group (S3A–S3C Fig). Following transfection, the MDMs were infected with Mtb H37Rv at a multiplicity of infection (MOI) of 1:5 (1 macrophage and 5 bacteria). miRNA-1246 inhibitor transfection significantly reduced Mtb growth in MDMs compared with that in the control group (Fig 2C). These results suggest that the inhibition of miRNA-1246 reduces Mtb growth in MDMs.

### miR-1246 regulates lipase activity in MDMs

miRNANAs regulate macrophage immune defense mechanisms by modulating the expression of genes involved in inflammation, pathogen recognition and immune responses [30–32]. To investigate the role of miRNA-1246 in regulating macrophage functions, MDMs were transfected with negative control oligonucleotides, an miRNA-1246 inhibitor, or a mimic in the presence or absence of Mtb H37Rv infection. Proteomic analysis was performed on the transfected MDMs after 72 hours using liquid chromatography-tandem mass spectrometry (LC-MS/MS) (Fig 3A and 3E). We identified ~4,045 proteins across all samples with high confidence (false discovery rate <1%), each supported by multiple peptide matches. As shown in S4A Fig, inhibition of miRNA-1246 in uninfected MDMs led to the upregulation of 142 proteins and the downregulation of 29 proteins compared with control uninfected MDMs transfected with negative oligonucleotides. Pathway analysis revealed that the inhibition of miRNA-1246 in uninfected MDMs increased the expression of molecules associated with apoptosis (S4C and S4D Fig), which is an important defense mechanism for controlling Mtb growth [33].

**Fig 3:**
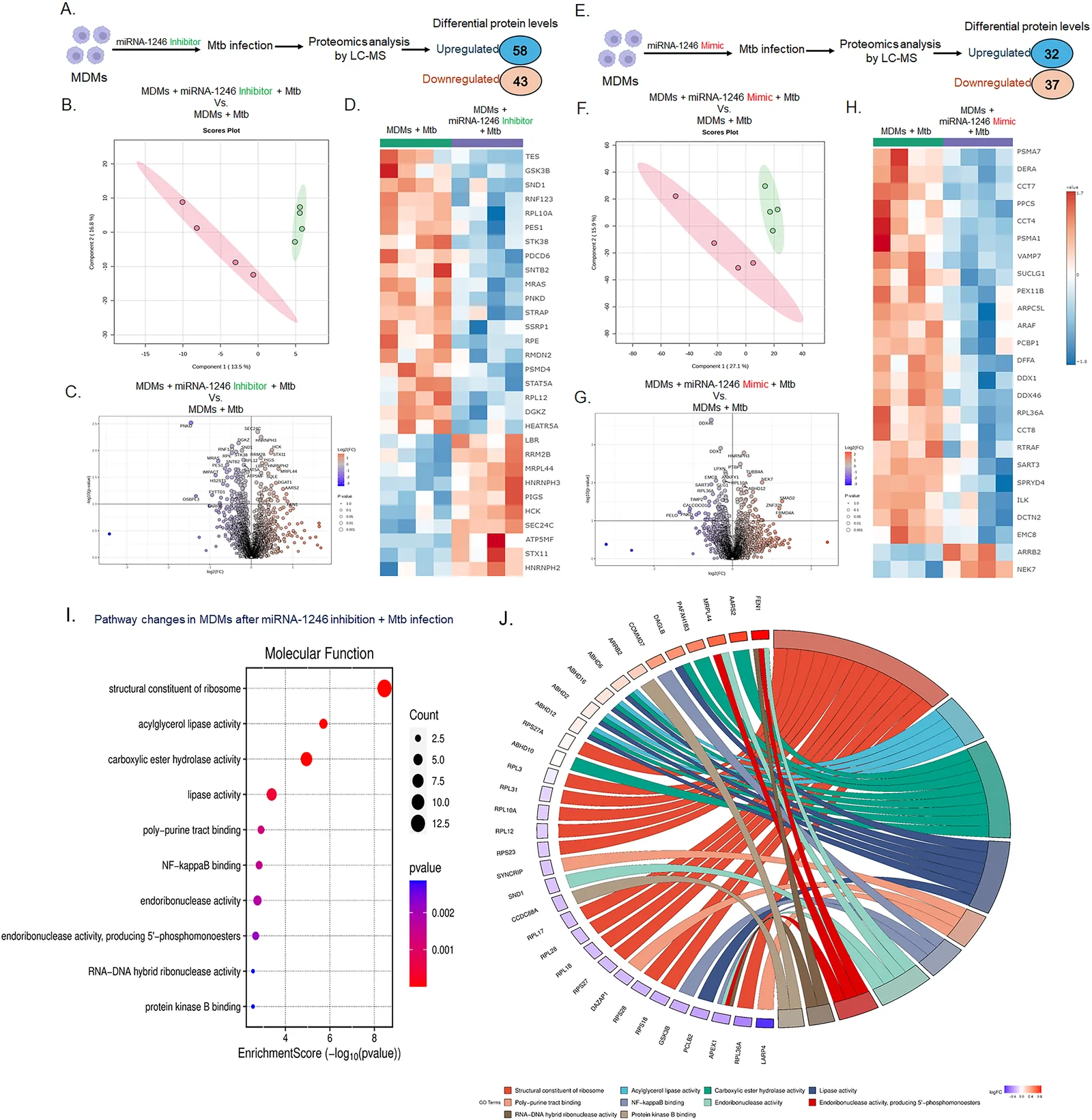
Proteomic analysis of miR-1246 regulation in Mtb-infected macrophages. MDMs were transfected with negative control oligonucleotides, miRNA-1246 inhibitor, or mimic, followed by Mtb H37Rv infection, and proteomic analysis using liquid chromatography-tandem mass spectrometry (LC-MS/MS) after 72 hours. **(A)** Timeline diagram of transfection of miRNA inhibitor followed by Mtb infection and number of regulated proteins are shown. **(B)** PCA plot distinguishes both the group. **(C)** Differential protein level in Mtb-infected MDMs transfected with miRNA-1246 inhibitor versus control oligos. **(D)** Heatmap shows top 30 significantly differentially expressed proteins. Each sample has 4 biological replicates represented by 4 columns in each sample (total 8 columns for two samples). **(E)** Timeline diagram of transfection of miRNANA mimic followed by Mtb infection. **(F)** PCA plot distinguishes both the group. **(G)** Differential protein levels in Mtb-infected MDMs transfected with miRNA-1246 mimic versus control oligos. **(H)** Heatmap shows top 30 significantly regulated candidates. Each sample has 4 biological replicates represented by 4 columns in each sample (total 8 columns for two samples). **(I)** Pathway analysis of protein enrichment in Mtb-infected MDMs transfected with miRNA-1246 inhibitor. **(J)** Chord plot showing the enrichment of proteins related to various biological activities in miRNA-1246 inhibitor-transfected Mtb-infected MDMs. Created in BioRender. Tripathi, D. (2026) https://BioRender.com/9ku33q4.

Mtb infection significantly altered the proteomic profile of MDMs (S5A Fig). In control oligo-transfected MDMs, infection led to increased expression of NFKB2, ICAM1, TRAF1, and other immune response-related proteins compared with that in uninfected controls (S5B Fig), which has previously been described as critical for defense against Mtb [34–36]. Additionally, Mtb infection significantly affected proteins involved in membrane targeting and ER protein trafficking (S5C Fig). Compared with control oligo-transfected Mtb-infected MDMs, MDMs transfected with the miRNA-1246 inhibitor and infected with Mtb presented the upregulation of 58 proteins and the downregulation of 43 proteins upon Mtb infection (Fig 3A–3C). miRNA-1246-inhibitor-transfected Mtb-infected MDMs presented decreased expression of GSK3B, STAT5A, PNKD, and RPL12, whereas SEC24C, PIGS, HCK, and HNRNPH3 were upregulated (Fig 3D). In contrast, miRNA-1246 mimic-transfected Mtb-infected MDMs presented downregulation of 37 proteins and upregulation of 32 proteins (Fig 3E–3H). Pathway analysis demonstrated that miRNA-1246 inhibitor-transfected Mtb-infected MDMs presented significantly higher enrichment scores for molecules associated with ribosomal structural constituents, acylglycerol lipase activity and lipase activity than control oligo-transfected Mtb-infected MDMs (Fig 3I). A clustered network enrichment test (CNET) chord plot further indicated that compared with Mtb-infected macrophages, miRNA-1246 inhibitor-transfected and Mtb-infected macrophages presented increased expression of lipase activity-related molecules (ABHD6, ABHD2, and ABHD16A), carboxylic ester hydrolase-related proteins (AARS2, PAFAH1B3, and DAGLB), and endoribonuclease-associated proteins (MRPL44 and FEN1) (Fig 3J). In summary, our findings suggest that miRNA-1246 in Mtb-infected macrophages regulates the expression of proteins involved in apoptosis and lipase activity, which are critical for restricting Mtb growth.

### miR-1246 target proteins are involved in acylglycerol metabolism

miRNAs regulate protein expression by binding to the 3’ untranslated region (UTR) of target mRNAs, which leads to mRNA degradation or the inhibition of translation [37,38]. We identified gene targets containing binding sites for miRNA-1246 within the 3’ untranslated region (UTR). We used TargetScanHuman Release 7.1 tool for miRNA target analysis and identified 3,043 potential targets of miRNA-1246 based on the presence of conserved 8-mer, 7-mer, and 6-mer seed match sites (Fig 4A).

**Fig 4:**
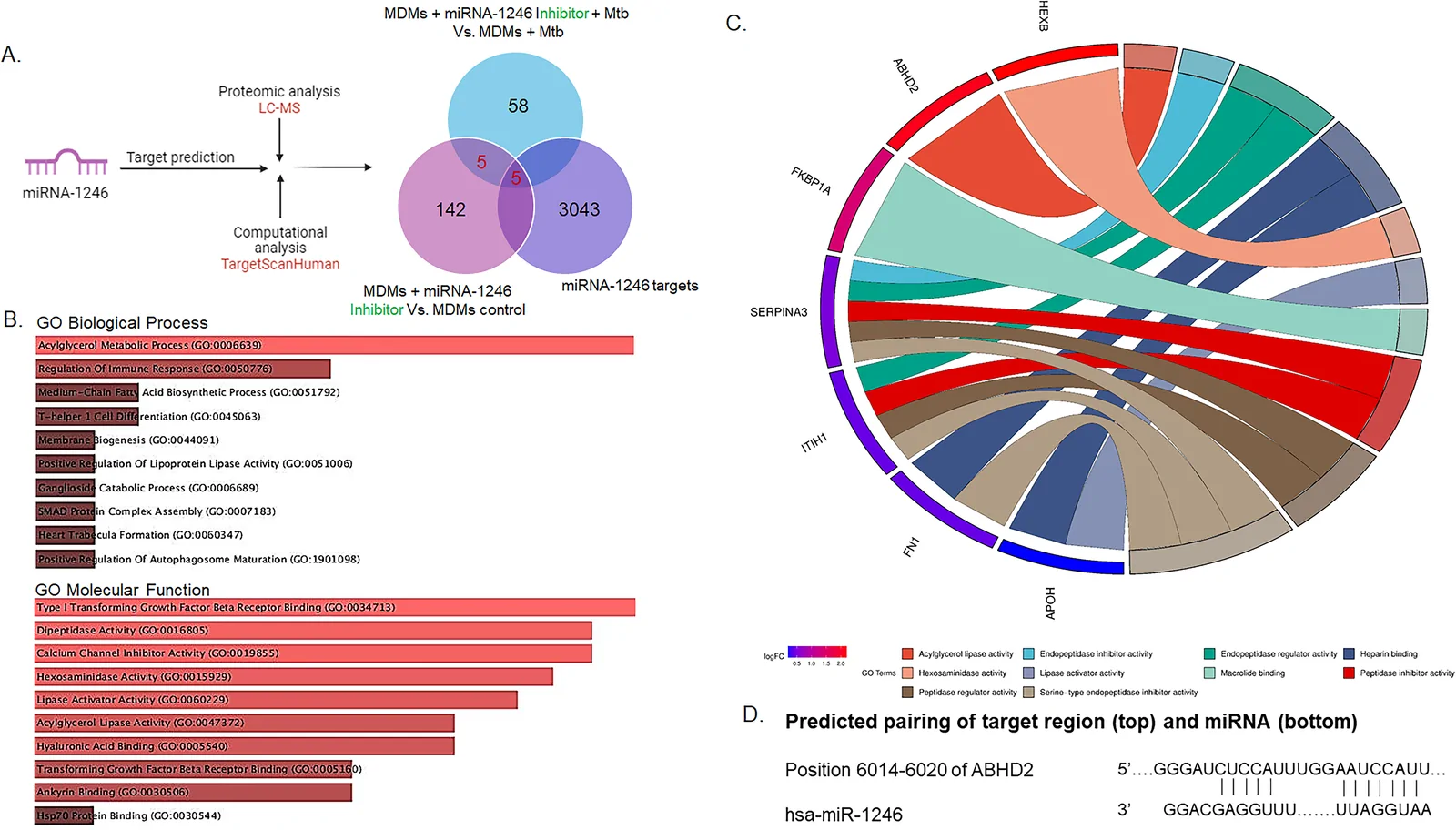
Identification and functional analysis of miR-1246 target proteins. **(A)** Computational prediction of miRNA-1246 target genes using TargetScanHuman Release 7.1 identified potential targets based on conserved seed match sites in the 3’ UTR (8-mer, 7-mer, and 6-mer) of mRNA with total predicted target number 3043 (purple sphere of venn diagram). The pink sphere shows 142 significantly upregulated protein candidates in macrophage transfected with miRNA-1246 inhibitor with respect to negative oligo transfection, similarly blue sphere shows 58 upregulated proteins in macrophages with miRNA-1246 inhibitor transfection along with Mtb infection with respect to only Mtb infection in macrophage samples. **(B and C)** Pathway enrichment analysis showing significant enrichment in acylglycerol metabolism and lipase activation (Gene Ontology analysis). **(D)** Computational prediction of proteomic target of miRNA-1246, which binds to a conserved seed sequence within its 3’ untranslated region (3’UTR). Created in BioRender. Tripathi, D. (2026) https://BioRender.com/wg9y682.

Next, we validate the identified targets by analyzing the proteomics data presented in Fig 3. As expected, transfection of the miRNA-1246 inhibitor into MDMs led to the upregulation of miRNA-1246 target proteins. We specifically focused on the proteins that were upregulated in miRNA-1246 inhibitor-treated MDMs. A total of 142 proteins were upregulated in uninfected MDMs transfected with the miRNA-1246 inhibitor compared with control oligo-transfected uninfected macrophages (Figs 4A and S4A). Additionally, 58 proteins were upregulated in Mtb-infected MDMs transfected with the miRNA-1246 inhibitor compared with control oligo-transfected Mtb-infected macrophages (Figs 3A and 4A). Seventeen proteins (DCTN6, ABHD2, FKBP1A, CPQ, HEXB, ST14, SPN, SPTBN1, ITIH1, SERPINA3, FN1, RAB7B, FYCO1, A1BG, APOH, IGHV4–34, and ARHGAP31) were commonly regulated (10 up- and 7 downregulated) in both uninfected and Mtb-infected miRNA-1246 inhibitor-transfected MDMs (Fig 4A and S1 Table). The above proteomic findings suggest that these 17 proteins could be potential targets of miRNA-1246.

To further investigate the significance and biological functions of these 17 target proteins of miRNA-1246, pathway enrichment analysis was performed. As shown in Fig 4B, gene ontology (GO) biological function analysis revealed significant enrichment in the acylglycerol metabolic process. GO molecular function analysis revealed enriched lipase activation and acylglycerol lipase activity. These findings suggest that miRNA-1246 may target proteins involved in lipase activity and the regulation of acylglycerol metabolism.

### Alpha/beta hydrolase domain-containing protein 2 (ABHD2) regulates lipid metabolism in MDMs and restricts Mtb growth

We next identified the proteins regulated by miRNA-1246 that are involved in acylglycerol metabolism. We conducted a clustered network enrichment test (CNET) analysis to identify the functional pathways associated with each protein. We found that ABHD2 was among the most significantly upregulated proteins among the 17 identified targets of miRNA-1246 and was associated with acylglycerol lipase activity (Fig 4C and S1 Table). ABHD2 was computationally predicted as a proteomic target of miRNA-1246, which binds to a conserved seed sequence within its 3’ untranslated region (3’UTR) (Fig 4A and 4D and S1 Table). To further validate these findings, we transfected MDMs from healthy controls with either a miRNA-1246 inhibitor or mimic. After 72 hours, qRT-PCR analysis showed that inhibition of miRNA-1246 significantly upregulated ABHD2 expression, whereas miRNA-1246 mimic treatment resulted in a significant reduction in ABHD2 expression (Fig 5A). These findings were further supported by proteomics data and confirmed by western blot analysis (Figs 5B and S6A and S6B). ABHD2 is known to be associated with lipase activity [39]. We quantified the neutral lipid content in MDMs using the BODIPY 493/503 green, fluorescent dye which binds neutral lipids following transfection with the miRNA-1246 inhibitor, mimic, ABHD2 siRNA, or control oligonucleotide. There was a significant reduction in neutral lipid content in MDMs transfected with the miRNA-1246 inhibitor compared with those transfected with the control oligo, whereas lipid accumulation was greater in MDMs transfected with the mimic or ABHD2 siRNA (Fig 5C and 5D). MDMs transfected with the miRNA-1246 mimic or ABHD2 siRNA showed significant increase in the Mtb growth. In contrast, Mtb growth was significantly low in macrophages transfected with the miRNA-1246 inhibitor (Fig 5E). To further determine whether Mtb growth depends on lipid accumulation in MDMs, we supplemented miRNA-1246 inhibitor transfected MDMs with oleic acid (OA; 200 µM, ~ 56 µg/mL) complexed to BSA (1%, ~ 10 mg/mL). After 72 hours, BODIPY staining revealed a marked increase in lipid content in miRNA-1246 inhibitor–transfected MDMs (Fig 5F and 5G). OA/BSA supplementation significantly increased Mtb CFU in miRNA-1246 inhibitor transfected MDMs compared to their non-supplemented Mtb infected MDMs (Fig 5H). However, no significant increase in neutral lipid accumulation or Mtb CFU was observed in miRNA-1246 mimic transfected MDMs after OA/BSA supplementation (S7A–S7C Fig). Our finding suggests that miRNA-1246 controls lipid content in MDMs by modulating ABHD2 expression, thereby controlling Mtb growth.

**Fig 5:**
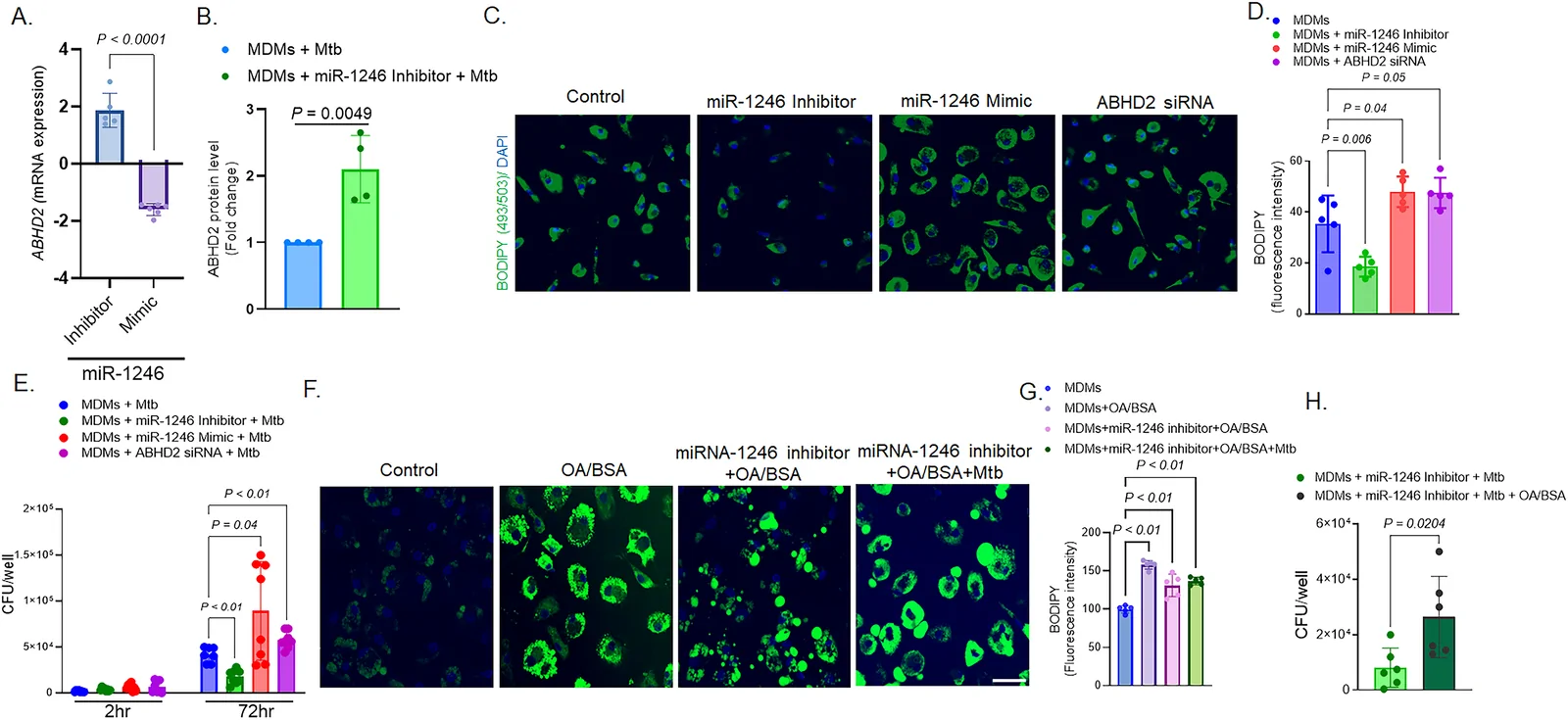
Impact of ABHD2 on lipid metabolism and Mtb growth in macrophages. **(A)** MDMs from healthy controls were transfected with either a miRNA-1246 inhibitor or mimic or negative control oligos. After 72 hours, quantitative real-time PCR analysis was performed to quantify ABHD2 expression at RNA level (n = 5-6/group). Data shown as mean ± SD and the *P*-value shown in each graph were determined by using a 2-tailed unpaired t-test. **(B)** Level of ABHD2 at protein level in miRNA-1246-inhibitor transfected and Mtb infected macrophages with respect to only Mtb infected macrophages, measured by LC/MS-MS (n = 4/group). **(C)** MDMs were transfected with miRNA-1246 inhibitor, mimic, ABHD2 siRNA or control oligonucleotide and stained with BODIPY (493/503) to quantify neutral lipid content. **(D)** Fluorescent intensity of BODIPY (493/503) was quantified from total 5 replicates (n = 5) for each samples using Fiji software. Statistical significance was determined by one-way ANOVA with Dunnett’s post hoc test. Data represents SD. P-values <0.05. **(E)** MDMs were transfected with miRNA-1246 inhibitor, miRNA-1246 mimic, ABHD2 siRNA, or control oligonucleotides, followed by infection with Mtb (MOI 1:1). Mtb growth was then assessed at 2 and 72 hours post-infection (n = 8/group). Statistical analysis was performed using Brown–Forsythe and Welch one-way ANOVA with Welch’s correction. Data are presented as mean ± SD. p < 0.05. **(F)** MDMs were transfected with miRNA-1246 inhibitor and treated with or without OA/BSA in combination with or without Mtb infection and stained with BODIPY (493/503) to quantify neutral lipid content. **(G)** Mean intensity of BODIPY (493/503) was quantified from total 4 biological and 5 technical replicates for each sample, using Fiji, ImageJ software. Statistical significance was determined by one-way ANOVA with Dunnett’s post hoc test. Data represents SD. *P-*values <0.05. **(H)** MDMs transfected with the miRNA-1246 inhibitor, treated with OA/BSA and infected with Mtb (MOI 1:1), at 72h Mtb growth was determined (n = 6/group). The *P*-value in each graph was calculated using an unpaired two-tailed t-test.

### Inhibition of miR-1246 induces glycolysis

During Mtb infection, the metabolic profile of macrophages skewed towards glycolysis and induce antimicrobial response to clear pathogen [40,41]. To understand the role of miRNA-1246 in regulating ABHD2 expression and its effects on metabolic pathways, we examined its impact on glycolysis in MDMs. MDMs were transfected with either a miRNA-1246 inhibitor or mimic and cultured in the presence or absence of the γ-irradiated Mtb. After 72 hours, miRNA-1246 inhibitor-transfected MDMs and miRNA-1246 mimic-transfected MDMs stimulated with γ-irradiated Mtb exhibited significantly enhanced extracellular acidification rate (ECAR) parameters such as glycolysis and increased glycolytic capacity compared with those transfected with control oligonucleotides (Fig 6A and 6B). miRNA-1246 mimic-transfected MDMs stimulated with γ-irradiated Mtb presented an inhibited glycolytic reserve. These results suggest that miRNA-1246 may play an important role in regulating macrophage metabolism during Mtb infection.

**Fig 6:**
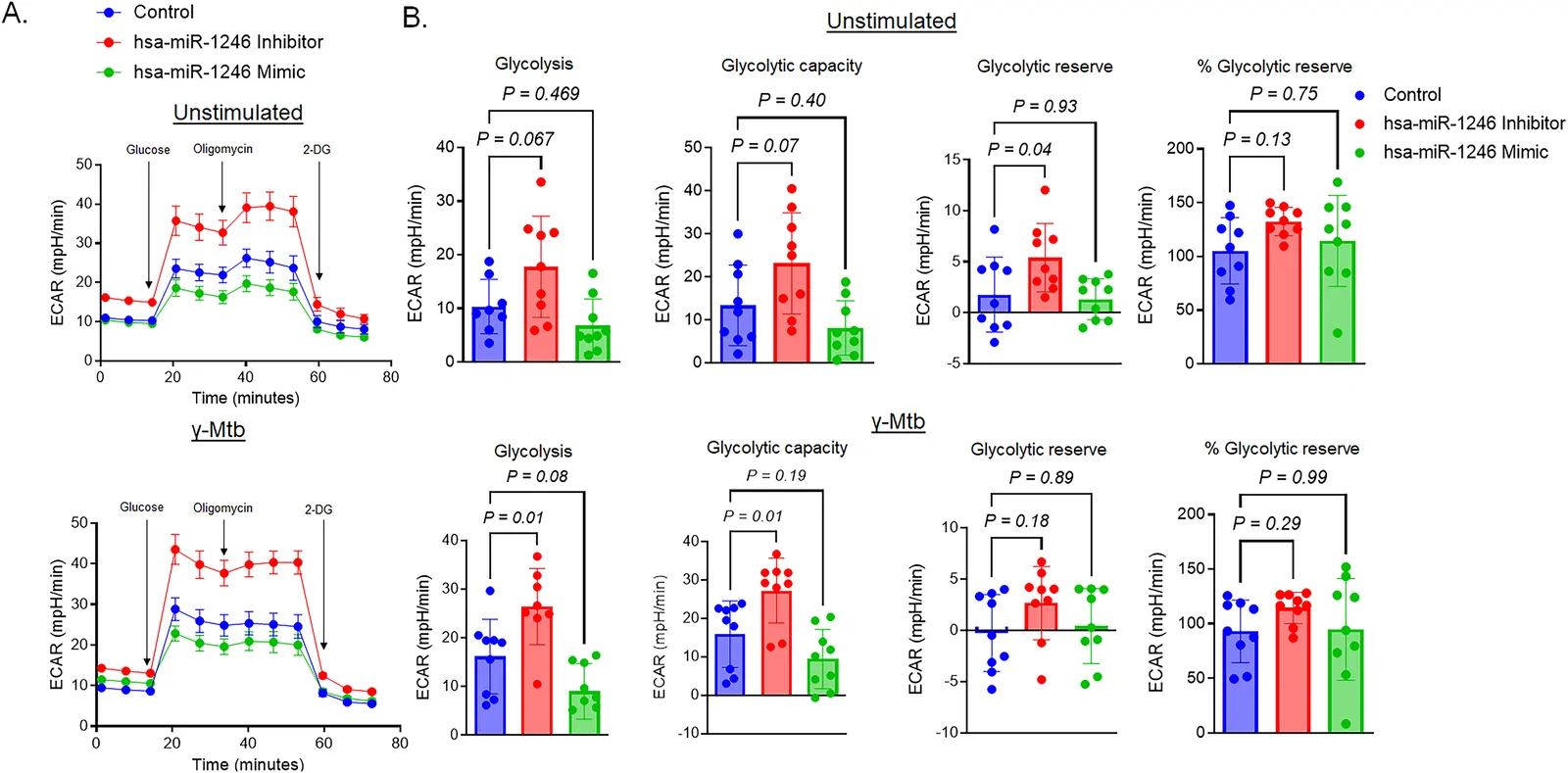
miR-1246 regulates macrophage bioenergetics. Human MDMs were transfected with either a miRNA-1246 inhibitor, mimic, or control oligonucleotides. After 24 hours, cells were stimulated with γ-irradiated Mtb or remain unstimulated for 48 h (total 72 h after transfection). **(A-B)** Extracellular acidification rate (ECAR) and glycolytic capacity were measured to assess glycolysis, for unstimulated (top panel) and γ-Mtb stimulated (bottom panel) MDMs. All the data is normalized by basal glycolysis (3^rd^ point of measurement) for each sample. Data are representative of three independent experiments. Statistical significance was determined by one-way ANOVA with Dunnett’s post hoc test. Data represents SD. *P-*values <0.05.

## Materials and methods

### Ethical statement

The study was approved by the institutional ethics committees of Bhagwan Mahavir Medical Research Center (BMMRC) and Blue Peter Public Health and Research Centre- LEPRA Society (BPHRC) in Hyderabad and written informed consent was obtained from each participant.

### Cohort description

A well-defined cohort of household contacts of pulmonary index TB patients was established as a part of the Regional Prospective Observational Research for Tuberculosis (RePORT)-India study in Hyderabad, India. Individuals (adults or children) residing in the same house as the index case for a minimum of 3 months prior to the date of the diagnosis of active TB were defined as HHCs. A total of 990 HHCs were included in this study, as mentioned in a previous report [20]. Out of 990, only 460 were IGRA negative at baseline (time of enrollment) and further followed in this study. These individuals shared at least 5 meals per week with the index case and had no history of TB or anti-tuberculosis treatment (ATT). All participants were screened for diabetes and HIV infection (by ELISA). HHCs with HIV, diabetes, pregnancy, autoimmune diseases or any other immunosuppressive conditions were excluded during screening. HHCs were recruited and assessed for either TB infection or disease at the time of enrollment as well as during the follow-up period (every 4 months for 2 years). Samples were compared between HHCs who remained IGRA negative (LTBI- or nonconverters) and those who developed LTBI or progressed to active TB (converters or LTBI+ and ATB progressors, respectively).

### Isolation and culture of PBMCs and CD14 + monocyte-derived macrophages (MDMs)

PBMCs were isolated by density gradient centrifugation using Ficoll‒Hypaque. Whole blood diluted with RPMI was layered over an equal volume of Ficoll and centrifuged for 30 min at 2000 RPM without braking. PBMCs were washed two times with PBS and counted by trypan blue staining. Freshly isolated PBMCs were cultured in 24 well plates at 1X10^6^ cells/well in RPMI-1640 with 1% penicillin/streptomycin (Sigma), L-glutamine and 10% heat inactivated human serum with or without CFP-10 and ESAT-6 (10 μg/mL) or γ-irradiated Mtb (10 µg/mL) at 37 °C in a humidified atmosphere with 5% CO_2_. MDMs were isolated with positive selection of CD14 + monocytes by using anti-human CD14 Micro Beads (Miltenyi Biotec, USA) according to the manufacturer’s protocol, and MDMs were derived using a standardized protocol as described previously [42].

### Preparation of the total RNA library and sequencing

Total RNA was obtained from unstimulated and CFP10 + ESAT6 stimulated cells (BEI resources, USA). Total RNA from the stimulated PBMCs of nonconverters, converters and progressors were extracted using TRIzol reagent. The RNA was quantified, and purity was assessed by using a 2100 Bioanalyzer (Agilent Technologies). A sequencing library was prepared by using 1 μg of total RNA for each sample by using the Illumina TruSeq RNA Sample Preparation Kit. In brief, the total RNA sample was treated with the Ribo-Zero rRNA Removal Kit (Epicenter, Madison, WI, USA) to deplete bacterial and eukaryotic ribosomal RNA (rRNA). The remaining RNA was converted to single-stranded cDNA using reverse transcriptase and random hexamers, and the second strand was synthesized to produce double stranded cDNA for use in TruSeq library construction using DNA polymerase I and RNase H. Then, these cDNA fragments underwent an end repair process. These cDNA were purified and enriched with PCR to create the final cDNA library. The libraries were counted and checked using a qPCR quantification protocol guide (KAPA Library Quantification Kits) and TapeStation D1000 ScreenTape (Agilent Technologies), respectively. Then cDNA libraries were sequenced using the HiSeq2500 platform (Illumina), which generated approximately 453 million paired end reads of 101 nucleotides in length.

### Transcriptome and bioinformatics analysis

Methods for data normalization and analysis are based on the use of “internal standards” that characterize some aspects of the system’s behavior, such as technical variability, as presented elsewhere [43,44]. These standards were initially created for the analysis of microarray data and were slightly modified to be applicable to RNA-seq data analysis. Differential gene expression analysis included the following steps. 1. The ‘reference group’ was constructed by identifying a group of genes expressed above background with inherently low variability as determined by an F test. The ‘reference group’ represents an internal standard of equal expression. As such, the ‘reference group’ is used to assess the inherent variability resulting from technical factors alone (technological variation). By creating an estimate of the technological variation, we were able to select a group of biologically stable genes. 2. Replications were selected using the commonly accepted significance threshold of FDR, *P* < 0.05 with Student’s t test. This selection maintains the commonly accepted sensitivity level; however, a significant proportion of genes identified as differentially expressed at this threshold will represent false-positive determinations. 3. An associative t test in which the replicated residuals for each gene from the experimental group are compared with the entire set of residuals from the reference group defined above. The Ho hypothesis was used to determine whether the levels of gene expression in the experimental group presented as replicated residuals (deviations from the average control group profile) were associated with a highly representative (several hundred members) normally distributed set of residuals of gene expression values in the reference group. The significance threshold was then corrected to render the appearance of false-positive determinations improbable: in the current case, the threshold was set at p < 0.0001. Only genes that passed both tests are presented in the final selections. Additional restrictions were applied to the minimal gene expression level (RPKM>2) and fold change (>1.5). The two-step normalization procedure and the associated analysis functions were implemented in MATLAB (Mathworks, MA). The raw fastq files, analyzed files, and metadata files were deposited in the NCBI Gene Expression Omnibus database (GEO GSE211566).

### TaqMan-qRT‒PCR

For RT‒qPCR analysis, total RNA was extracted from preserved PBMCs from nonconverters, converters and progressor individuals using TRIzol reagent (Invitrogen, USA). For cDNA synthesis, a total of 20 ng of RNA was used for cDNA synthesis using a TaqMan Advanced miRNA Assay Kit (Thermo Fisher Scientific, USA) according to the manufacturer’s protocol. Briefly, cDNA synthesis involves four different steps. First, a poly (A) tailing reaction was performed on RNA in a total 5 µl reaction, followed by second steps of adapter ligation in a 15 µl final reaction volume by using RNA ligase enzyme and ligation adapter mix. In the third step, reverse transcription was performed by using 20x Universal RT primers and RT enzyme mix in a reaction volume of 30 µL at 42 °C for 30 minutes. Finally, in the fourth step, the miRNA-Amp reaction was performed by using 2x miRNA-Amp Master Mix and 20X miRNA-Amp Primer Mix in a total volume of 50 µl.

TaqMan-based real-time PCR was performed by using TaqMan Fast Advance Master Mix and a TaqMan Advance miRNA assay (20x) prepared for a suitable number of reactions. Real-time PCR was run on a QuantStudio 7 Flex System, and the data were analyzed by the ΔΔCt method to calculate the fold change and ΔCt for the relative copy number (RCN). miRNA-191 was used as a housekeeping reference gene for the ΔCt calculations. Comprehensive list of miRNAs, primers and their corresponding sequences utilized in this study are provided in S2 Table.

### CFU analysis in MDMs

A total of 40000 CD14 + -derived MDMs were seeded in 96-well tissue culture plates, infected with Mtb at a multiplicity of infection (MOI) of 1:5 or 1:1 and incubated for 2 h at 37 °C in a CO_2_ incubator. After 2 h of infection, the extracellular Mtb was removed by washing three times with warm PBS. To enumerate CFUs at the indicated time points, infected MDMs were lysed with 0.06% v/v SDS, and suitable serial dilutions were plated on 7H10 agar plates. CFUs were counted after three weeks of incubation.

### Transfection of miRNA mimics and inhibitors

The mimics and inhibitors were transfected with Lipofectamine RNAiMAX reagent (Thermo Fisher, USA) according to the manufacturer’s standard protocol. Finally, 1 pmol of mimics/inhibitor/siRNA and 0.3 µl of Lipofectamine diluted in an equal volume of Opti-MEM medium was used per well of a 96-well plate with 40000 seeded CD14 + MDMs. Scrambled RNA alongside Lipofectamine and Opti-MEM reaction premix were used as a negative control. Twenty-four hours after transfection, the cells were used for Mtb infection or Seahorse XF experiments for measurement of the ECAR and BODIPY staining.

### Lipid supplementation in miR-1246 inhibited macrophages

MDMs were transfected with a miRNA-1246 inhibitor or corresponding control using standard transfection protocols described above. To assess whether lipid availability influences Mtb growth, cells were supplemented with oleic acid (OA) complexed to bovine serum albumin (BSA). Briefly, OA was prepared at a final concentration of 200 µM (~56 µg/mL) and conjugated to 1% (w/v) fatty acid–free BSA (~10 mg/mL) to enhance solubility and cellular uptake. The OA-BSA complex was added to culture media immediately following transfection. MDMs were incubated with OA–BSA for 48 hours under standard culture conditions (37°C, 5% CO_2_). Following supplementation, intracellular lipid accumulation was assessed using BODIPY 493/503 staining.

### BODIPY staining

We quantified the neutral lipid content in MDMs using the BODIPY 493/503 green, fluorescent dye which binds neutral lipids following transfection with the miRNA-1246 inhibitor, mimic, ABHD2 siRNA or control oligonucleotide and after OA/BSA supplementation. Cells were washed with PBS, fixed with 2% paraformaldehyde for 15 minutes at room temperature, and subsequently stained with BODIPY (1 µg/mL in PBS) for 30 minutes in the dark. After washing, nuclei were counterstained with DAPI and cells were imaged using fluorescence microscopy. Quantitative analysis of lipid content within each individual cell was performed by measuring BODIPY fluorescence intensity [45,46].

### Liquid chromatography-tandem mass spectrometry (LC-MS/MS)

Four biological samples were prepared with or without 48 h post Mtb infection of CD14 + MDMs in combination with 24 h (total 72 h) of pre-transfection with the miRNA mimic, inhibitor or negative control. Then 6 MDM samples (transfected with the control, miRNA-1246 inhibitor or mimic with or without Mtb infection) were prepared from four individual donors (24 samples). Protein samples were extracted and sent for LC‒MS/MS-based analysis of peptide quantification in proteomics core facility in UT southwestern, Dallas.

Protein samples were analyzed on a high-resolution liquid chromatography tandem mass spectrometry (LC-MS-MS) system. An “Ultimate 3000 RSLCnano system coupled to a Q Exactive HF-X mass spectrometer was used. Samples were desalted and concentrated on a C18 precolumn at a flow rate of 5 µL/min. Peptides were then separated on a C18 analytical column through a linear gradient of 5–35% acetonitrile with 0.1% formic acid over 120 minutes at a flow rate of 300 nL/min. The mass spectrometer was operated in data dependent acquisition mode, with a full MS scan range of 350–1600 m/z at a resolution of 60,000, followed by MS/MS scans of the top 20 most intense ions.

Quality control was done to confirm data reproducibility and instrument performance. A standard reference sample was evaluated at the beginning, middle, and end of each run. Key performance metrics, including peptide identification rates, retention time stability, and signal intensity, were monitored. The mass accuracy was maintained within 5 ppm, and samples with less than 80% peptide identification consistency compared with the reference were flagged for reanalysis. Also, blank runs were performed between samples to minimize carryover, and system suitability was verified using a 6-protein digest standard (Pierce 6 Protein Digest).

### Data processing and mapping

LC-MS/MS data were processed using MaxQuant for peptide detection and quantification. The spectra were searched against the UniProt human reference proteome. Identified proteins were annotated using Gene Ontology (GO) terms and Kyoto Encyclopedia of Genes and Genomes (KEGG) pathways by the DAVID bioinformatics tool. Statistical analysis of differential expressions was conducted using Perseus, applying a two-sample t test with a *P-*value threshold of 0.05. For each sample, ~ 4045 peptides were identified with high confidence. The data were analyzed using Proteome Discoverer 2.4 and were searched against the human protein database from UniProt.

### Glycolysis flux assay

Extracellular acidification rate was measured using a Seahorse XF96 extracellular flux analyzer (Agilent). MDMs were seeded into the XF96 cell culture plate at a density of 100,000 cells/well. MDMs were differentiated directly in XF96 cell culture plates. Furthermore, glycolytic stress in CD14 + MDMs was measured with and without stimulation with γ-irradiated Mtb in addition to mimic, inhibitor or negative control transfections. The Wave Desktop 2.6 Software, available from the Agilent website (https://www.agilent.com/en/products/cell-analysis/software-down-load-for-wave-desktop), was used to normalize the ECAR values and export the data into GraphPad Prism Version 7.04 for statistical analysis. The Wave Desktop 2.6 software also provides the capability to export the data into the XF Report generators for calculation of the parameters from the extracellular acidification assay (Glycolysis Stress Test).

In the extracellular acidification test, the first three basal ECAR readings (2 min of mixing and 3 min of measurement) were used to measure nonglycolytic acidification. After the addition of 10 mM glucose, three ECAR measurements were used to determine the extent of extracellular acidification resulting from glucose metabolism after subtraction of the nonglycolytic acidification. This was followed by the addition of oligomycin to inhibit ATP synthase in the mitochondria, forcing the cell to use glycolysis solely to generate all the ATP demands of the cell. Three subsequent ECAR measurements provided a measure of the maximal glycolytic capacity after subtraction of the nonglycolytic acidification. The difference between the glycolytic capacity and the ECAR from glucose metabolism provides the glycolytic reserve, which is a measure of the ability of a cell to respond to energetic demands, in addition to indicating how close the ECAR from glucose metabolism is to the theoretical maximum of the cell. Glycolysis was then inhibited with the addition of 2.5 mM 2-deoxyglucose (2-DG), and three subsequent ECAR measurements demonstrated that the prior ECAR resulted from glycolysis, hence providing a measure for nonglycolytic acidification.

### Western blot analysis of ABHD2 protein

For ABHD2 protein analysis, CD14 ⁺ monocyte-derived macrophages (MDMs) were transfected with negative control oligonucleotides, miRNA-1246 inhibitor, miRNA-1246 mimic, or ABHD2-specific siRNA and incubated for 72 h. Following transfection, cells were washed with 1X HBSS and lysed using RIPA lysis buffer (Thermo Scientific; #89901) supplemented with a protease and phosphatase inhibitor cocktail (Thermo Scientific; #1861280). Protein concentrations were determined, and ~20 µg of total protein per sample was resolved by SDS-PAGE and subsequently transferred onto nitrocellulose membranes. Membranes were blocked and incubated overnight at 4 °C with primary antibodies against ABHD2 (Sigma-Aldrich; #SAB2103165) or β-actin (Cell Signaling Technology; #4970L). After washing, membranes were incubated with HRP-conjugated secondary antibodies (Cell Signaling Technology, #7074S). Protein bands were visualized using chemiluminescence method according to the manufacturer’s instructions (Bio-Rad; Western ECL Substrate #1705062). ABHD2 protein was detected at molecular weight of approximately ~23 kDa, with β-actin (~42 kDa) used as the loading control.

### Statistical analysis

All statistical analyses were performed using GraphPad Prism version 10.0 (GraphPad Software, San Diego, CA, USA). Descriptive analysis was performed for all relevant variables prior to their inclusion in the analysis. Frequency counts and percentage were obtained for internal and ordinal categorical data. *P*values less than 0.05 were considered statistically significant. Data are presented as mean ± standard deviation (SD). Comparisons between two independent groups were performed using a two-tailed unpaired Student’s t-test. Comparisons among three or more groups were analyzed using one-way analysis of variance (ANOVA) followed by Tukey’s or Dunnett’s multiple-comparisons test, as appropriate. When the assumption of equal variances was not met, Brown–Forsythe and Welch one-way ANOVA followed by Welch’s multiple-comparisons test was applied.

## Discussion

We determined the role of miRNAs in resistance to the development of latent or active TB in the HHCs of TB patients. We followed 460 HHCs at regular intervals (0, 4, 8, 12, 16, 20 and 24 months) and found that 293 never developed latent or active TB [20]. In the present study, we compared the miRNA profiles of nonconverters (those who never developed latent or active TB within 2 years) and those converted to LTBI or active TB progressors. MicroRNA profiling, confirmation experiments and macrophage function studies demonstrated that low expression of miRNA-1246 in nonconverters at baseline (“0” time point) and follow-up (24 months) compared with progressors and converters is involved in protection against the development of latent or active TB. Decreased miRNA-1246 expression leads to reduced Mtb growth in MDMs through decreased activity of the lipase ABHD2 and increased glycolytic activity. Overall, our studies demonstrate that miRNA-1246-dependent mechanisms are involved in resistance to Mtb infection in the HHCs of TB patients.

miRNAs are small RNA molecules that regulate the transcription of genes and affect critical biological functions, including immune responses to infections [5]. During Mtb infection, miRNAs modulate immune responses, including the activation of macrophages and T cells, which are crucial for controlling the establishment of Mtb infection [18,47]. However, the role of miRNAs in the resistance to or development of latent or active TB is not known. In the present study, principal component analysis (PCA) distinguished nonconverters from both LTBI converters and active TB progressors in the miRNA profile. We identified 28 upregulated and 10 downregulated miRNAs in the PBMCs of Mtb antigen-stimulated cells during baseline and follow-up in resisters compared with those having latent or active TB. Among these miRNAs, miRNA-1246 expression is decreased, and miRNA-125b expression is increased. The role of miRNA-125b during Mtb infection is known, and we focused on the relevance of decreased expression of miRNA-1246 and resistance to Mtb infection. Our real-time PCR validation experiments confirmed our miRNA analysis findings.

miRNA-1246 has been linked to various inflammatory diseases and infections. It regulates immune responses and inflammation by targeting key signaling pathways, such as the NF-κB and TNF-α pathways, and can regulate the lipid signaling pathway in intracranial aneurysms [48]. miRNA-1246 modulates the host immune system by regulating IL-6 in macrophages, with elevated levels observed in rheumatoid arthritis [49]. Interestingly, the HCV/HIV coinfection downregulates miRNA-1246, suggesting that it may play a role in increased susceptibility to infections [50]. MicroRNA-1246 plays a significant role in regulating macrophage polarization and function, influencing immune responses in disease models and metabolic diseases [51]. No information is available on the role of miRNA-1246 during Mtb infection. The inhibition of miRNA-1246 in MDMs led to a significant reduction in Mtb growth (Figs 2C and 5E), suggesting that miRNA-1246 negatively regulates key macrophage functions essential for the control of Mtb infection. miRNANA-1246 is known to target a variety of genes involved in cancer progression, immune responses, cell proliferation, migration and apoptosis [22–24,29,52]. Proteomic analysis of Mtb-infected and uninfected MDMs transfected with the miRNA-1246 inhibitor revealed alterations in proteins linked to key immune pathways, including apoptosis and lipase activity. miRNA-1246 inhibition enhanced lipase activity in MDMs, which was associated with reduced lipid accumulation and increased restriction of Mtb growth. These findings suggest that miRNA-1246 plays a critical role in modulating macrophage metabolism during Mtb infection, potentially influencing the host’s ability to control bacterial growth through lipid metabolism.

Host lipids serve as a critical energy source for the growth and survival of Mtb [53,54]. This is the first report to reveal a novel function of miRNA-1246. Inhibition of miRNA-1246 led to the upregulation of acylglycerol lipase activity, which is involved in the breakdown of lipid molecules. Enhanced lipase activity may thus limit Mtb growth by depriving the bacteria of necessary lipids, suggesting a novel immune mechanism regulated by miRNA-1246. Pathway enrichment analysis of proteins regulated by miRNA-1246 in Mtb-infected MDMs revealed that ABHD2 is a critical target of miRNA-1246. ABHD2 is involved in acylglycerol metabolism and lipase activity [39] and is upregulated in MDMs treated with a miRNA-1246 inhibitor. Compared with control macrophages, macrophages transfected with the miRNA-1246 inhibitor presented increased ABHD2 expression and decreased Mtb growth. ABHD2 siRNA significantly increased Mtb growth in MDMs. The inhibition of miRNA-1246 and hence the upregulation of ABHD2 were associated with reduced lipid accumulation and a concomitant decrease in Mtb growth, suggesting an important role for miRNA-1246 in modulating ABHD2 expression and lipid metabolism to increase the ability of macrophages to restrict Mtb replication. ABHD2 is reported to be involved in metabolic diseases, as its deficiency in mice leads to emphysema due to disrupted phospholipid metabolism and increased inflammation [55,56]. Additionally, ABHD2 upregulation has been observed in cells transfected with hepatitis B virus DNA [57]. Specifically, in the lungs, ABHD2 plays a crucial role in the recruitment of monocytes and macrophages [58], highlighting its potential involvement in infectious diseases.

Macrophage metabolism significantly changes during Mtb infection, with a shift toward glycolysis to support the energy demands of immune activation [40,41]. Inhibition of miRNA-1246 in MDMs increased glycolysis and glycolytic capacity, suggesting that miRNA-1246 modulates bioenergetic pathways in macrophages. ABHD2 regulates macrophages via TGF-beta signaling [55], which, in turn, modulates macrophage glycolysis by increasing the expression and activity of the glycolytic enzyme PFKL. This glycolytic process is driven through a mTOR-CMYC-dependent pathway [59]. This metabolic reprogramming could provide an additional layer of defense against Mtb by ensuring the availability of energy for antimicrobial responses. The connection between lipid metabolism, glycolysis and macrophage function in the context of Mtb infection in the HHCs of TB patients highlights the complex interplay between metabolic pathways and immune responses. The ability of miRNA-1246 to regulate both lipid and glucose metabolism suggests that it plays a critical role in modulating macrophage immune function to restrict Mtb growth.

Metabolic rewiring of macrophages is a central determinant of Mtb control [60,61]. Mtb infection imposes a quiescent bioenergetic state in human monocyte-derived macrophages, characterized by reduced glycolytic and TCA cycle flux [41], a phenotype thought to favor bacterial persistence. In contrast, restoration or enhancement of glycolysis promotes antimicrobial activity, in part by driving pro-inflammatory effector functions such as IL-1β production, which is essential for Mtb control [62]. Notably, glycolytic end products such as lactate can further reinforce host defense through mechanisms including autophagy, highlighting a complex but overall beneficial role of glycolytic reprogramming in restricting bacillary survival [63,64]. In parallel, host lipid metabolism critically influences Mtb growth, as lipid droplets serve as nutrient reservoirs for the pathogen; however, dysregulated lipid droplet biogenesis can also limit bacterial replication depending on context, underscoring a finely tuned balance between host defense and pathogen exploitation [65–67]. Consistent with this, our data demonstrates that exogenous lipid loading via oleic acid/BSA supplementation significantly increased intracellular lipid accumulation and enhanced Mtb CFU in miRNA-1246 inhibitor transfected macrophages. Our findings demonstrate that elevated lipid availability promotes bacterial growth. We found that miRNA-1246 drives glycolytic activity while simultaneously restricting lipid accumulation through regulation of ABHD2, thereby counteracting the Mtb-induced metabolic quiescence and limiting lipid-dependent bacterial growth. Our findings demonstrate that miRNA-1246 is a key integrator of glycolytic and lipid metabolic pathways that coordinately regulate macrophage antimicrobial capacity against Mtb.

Overall, our study provides new insights into the mechanistic role of miRNA-1246 in resistance to Mtb infection. For the first time, our findings demonstrate that miRNA-1246 is involved in resistance to human Mtb infection and regulates macrophage responses by modulating lipid metabolism, apoptosis and bioenergetic pathways, thereby restricting Mtb growth. Given its involvement in key immune pathways, miRNA-1246 may represent a promising target for the development of new anti-TB therapies, particularly for HHCs, who are at high risk of developing latent or active TB. Furthermore, the differential miRNA profiles observed in nonconverters could be used as biomarkers for identifying individuals with natural resistance to TB. These biomarkers could help in developing personalized strategies for TB prevention and treatment, especially for HHCs of TB patients and lay the foundation for future therapeutic strategies targeting miRNAs to enhance host immunity against tuberculosis.

## Supporting information

S1 Fig Non-converters exhibit differential expression of miR-1246. PCA plot illustrates that based on 38 (28 up and 10 down-regulated) miRNAs non-converter sample can be distinguished from converters and progressors both the time point of baseline as well as follow up. Created in BioRender. Tripathi, D. (2026) https://BioRender.com/e7du35z.
(TIF)

S2 Fig Non-converters exhibit differential expression of miR-1246 and miR-125b miRNAs. (**A**) Shows flowchart for selection criteria of candidate miRNA miRNA-1246 and miRNA-125B2 out of high throughput data from more than 1900 miRNA sequenced from RNA seq. (**B**) qRT-PCR for the selected 8 miRNAs having significant expression below <40 CT values. miRNA-191 was used as an internal control for relative copy number (RCN) calculations for each miRNA. Data are presented as mean ± SD of three experiments.
(TIF)

S3 Fig Validation of miR-1246 transfection in MDMs. (**A**) Percentage inhibition of miRNA-1246 following transfection with miRNA-1246 inhibitor in human monocyte-derived macrophages (MDMs). (**B**) Percentage increase in miRNA-1246 expression following transfection with miRNA-1246 mimic in MDMs. For panels (**A**) and (**B**) miRNA-1246 expression was quantified by qRT-PCR at 24 and 48 h post-transfection and calculated relative to negative control oligonucleotide transfected cells. (**C**) Fold-change representation of miRNA-1246 expression derived from the same qRT-PCR data shown in panels (**A**) and (**B**), with negative control transfection set as the reference. miRNA-191 was used as the endogenous internal control for normalization. Data are presented as mean ± SD of three experiments.
(TIF)

S4 Fig Proteomic analysis of miR-1246 regulation in uninfected macrophages. MDMs were transfected with negative control oligonucleotides, miRNA-1246 inhibitor, and proteomic analysis using liquid chromatography-tandem mass spectrometry (LC-MS/MS) after 72 hours. (**A**) Timeline diagram of transfection of miRNA inhibitor and number of regulated proteins are shown. (**B**) Heatmap of significantly regulated protein candidates in miRNA-1246-inhibitor and negative control oligo transfected macrophages samples (each has 4 columns represent 4 biological samples). (**C**) Pathway enrichment analysis depending upon significantly up and down regulated proteins. (**D**) Volcano plots showing LC-MS based significant fold change (X-axis) of regulated proteins candidate in miRNA-1246-inhibitor with respect to negative control oligo transfected macrophages samples. Created in BioRender. Tripathi, D. (2026) https://BioRender.com/9ku33q4.
(TIF)

S5 Fig Identification and functional analysis of miR-1246 target proteins. (**A**) PCA plot illustrates that based on top 50 significantly regulated protein in LC-MS, un-infected samples can be distinguished from infected macrophages. (**B**) Volcano plots showing LC-MS based significant fold change (X-axis) of regulated proteins candidate in Mtb infected macrophages with respect to uninfected macrophages samples. Here Y axis is -log10(P-value). (**C**) Pathway enrichment analysis depending upon significantly up and down regulated proteins.
(TIF)

S6 Fig Western blot analysis of ABHD2. (**A**) Western blot analysis of ABHD2 from hMDMs transfected with negative control oligo, miRNA-1246 Inhibitor, Mimic and ABHD2 siRNA. One representative blot is shown here. (**B**) Intensity of ABHD2 expression is quantified from four independent biological donors. ABHD2 expression was normalized by β-actin expression for corresponding samples. (**C**) Corresponding full blot for S6 Fig A is shown.
(TIF)

S7 Fig Impact of OA/BSA supplementation on Mtb growth in miR1246-inhibited macrophages. (**A**) MDMs were transfected with miRNA-1246 mimic and supplemented with oleic acid and BSA in combination with or without Mtb infection and stained with BODIPY (493/503) to quantify neutral lipid content. (**B**) Mean fluorescent intensity (MFI) of BODIPY (493/503) was quantified from total 4 biological and 5 technical replicates (n = 4, n = 5 each) for each samples using Fiji software. (**C**) MDMs transfected with the miRNA-1246 inhibitor, mimic, ABHD2 siRNA, control oligos and some wells were supplemented with OA/BSA and infected with Mtb (MOI 1:1) at (72h) Mtb growth was determined. Data are representative of six independent experiments. The p-value shown in each graph was determined by one-way ANOVA for the selected group which is represented in line.
(TIF)

S1 Table Seventeen proteins commonly regulated in miR-1246 inhibitor–transfected MDMs under both uninfected and Mtb-infected conditions compared to their respective uninfected and Mtb-infected controls. (TIF)

S2 Table Comprehensive list of miRNAs, primers and their corresponding sequences utilized in this study. (TIF)
